# Supplementary material for: Chronic oxidative stress promotes H2AX protein degradation and enhances chemosensitivity in breast cancer patients
Source: EMBO Mol Med. 2016 Mar 22;8(5):527–49. doi: 10.15252/emmm.201505891 (PMC5123617; doi:10.15252/emmm.201505891)
Supplement: Supplementary file 1 — Appendix [file EMMM-8-527-s001.pdf]

APPENDIX

Chronic oxidative stress promotes H2AX protein degradation and enhances chemosensitivity in breast cancer patients

Gruosso Tina[1,2, §], Mieulet Virginie[1,2, §], Cardon Melissa [1,2], Bourachot Brigitte[1,2], Kieffer Yann [1,2], Devun Flavien[3], Dubois Thierry[4], Dutreix Marie[3], Vincent-Salomon Anne[5], Miller Kyle M.[6] and Mechta-Grigoriou Fatima[1,2*]

Running title: Role of H2AX down-regulation by ROS in cancer

TABLE OF CONTENT

Figure S1: Evaluation of DDR proteins in cells with chronic stress

Figure S2: *H2afx* mRNA, miRNA regulation of H2AX and impact of protein synthesis or proteasome inhibitors on H2AX protein levels.

Table S1: Semi-quantitative expression profiling of proteins involved in cell cycle and DNA damage pathway

Table S2: List of genes involved in oxidative stress response and up-regulated in low- *versus* high-H2AX TN breast tumours

Table S3: List of genes involved in oxidative stress response and up-regulated in low- *versus* high-H2AX HER2 breast tumours

Figure S1: Evaluation of DDR proteins in cells with chronic stress

A **Up**, Representative western blots from whole cell extract showing ter surgery.2AX protein levels in *wt* and *cat*$^{-/-}$ fibroblasts, after H_2O_2 exposure for the indicated times (hours, h). Actin is used as internal control for protein loading. **Down**, Bar plots showing γ-H2AX and H2AX protein levels, as well as the ratio γ-H2AX/H2AX as assessed by densitometry analysis of western blots (as shown above). Data are shown as means ± s.e.m.. NS stands for not significant and P-values are based on paired t-test showing the difference between *wt* and *cat*$^{-/-}$ fibroblasts.

B,C Bar-plots showing levels of several DNA-damage proteins in *wt* and *junD*$^{-/-}$ (**B**) and *Nfe2l2*$^{-/-}$ (**C**) fibroblasts, as assessed by Reverse Phase Protein Array (RPPA). Data are shown as means ± s.e.m. from n=3 independent experiments. NS stands for not significant and P-values are based on Student's t-test.

Figure S2: *H2afx* mRNA, miRNA regulation of H2AX and impact of protein synthesis or proteasome inhibitors on H2AX protein levels.

A Bar plots showing *H2afx* mRNA levels in *wt* and *junD*$^{-/-}$ fibroblasts, as assessed by qRT-PCR analysis.

B Bar plots showing miR-24 and miR-138 levels in *wt* and *junD*$^{-/-}$ fibroblasts, as assessed by qRT-PCR analysis.

C Bar plots showing the indicated *in silico* predicted H2AX-targeting miRNA levels in *wt* and *junD*$^{-/-}$ fibroblasts, as assessed by qRT-PCR analysis.

D Up, Representative western blot from whole cell extracts showing H2AX protein levels in *wt* and *junD*$^{-/-}$ fibroblasts, under untreated conditions (-) or after 8h of treatment (+) by the protein translation inhibitor Cycloheximide (CHX). Down, bar plots showing H2AX protein levels as assessed by densitometry analysis of western blots (as shown above).

E Up, Representative western blot from whole cell extracts showing H2AX protein levels in *wt* and *junD*$^{-/-}$ fibroblasts, under untreated conditions (-) or after 8h of treatment (+) by

proteasome inhibitor MG132. Down, Bar plots showing H2AX protein levels as assessed by densitometry analysis of western blots (as shown above).

F Up, Representative western blot from chromatin-free fraction or whole cell extracts, showing H2AX protein levels in *wt* and *Nfe2l2*$^{-/-}$ fibroblasts. Two different exposures (low or high) and amounts of loaded proteins are presented, H2AX being easier to detect when associated with chromatin. Down, bar plots showing H2AX protein levels as assessed by densitometry analysis of western blots (as shown above).

G Up, Representative western blot from chromatin-free fraction extracts showing H2AX protein levels in *wt* and *junD*$^{-/-}$ fibroblasts, under untreated conditions (-) or after 8h of treatment (+) by the protein translation inhibitor Cycloheximide (CHX). Down, Bar plots showing H2AX protein levels as assessed by densitometry analysis of western blots (as shown above).

H Representative western blots from whole cell extracts showing HA-tagged ubiquitin (Left) or Flag-tagged H2AX (Right) protein levels. *wt* and *junD*$^{-/-}$ fibroblasts were co-transfected with vectors encoding HA-tagged ubiquitin and Flag-tagged wild-type H2AX (FLAG-H2AX WT) or empty vector, as indicated. Flag-tagged H2AX proteins were then immunoprecipitated with Flag-specific antibody and incubated either with HA-specific antibody (Left) or Flag-specific antibody (Right).

I Bar plots showing mRNA levels of RNF168, RNF8 and BMI1 in junD-deficient fibroblasts after transfection of a siRNA control, or directed against the indicated protein.

For all panels, data are shown as means ± s.e.m. (n=3 independent experiments) NS stands for not significant and P-values are based on Student's t-test. Jun is used as positive control for treatment efficiency (D,E,G). Actin is used as internal control for protein loading (D-G).

Table S1: Semi-quantitative expression profiling of proteins involved in cell cycle and DNA damage pathway

Results are shown as fluorescent intensity means (each antibody being coated in sixplicates on the slides) after hybridization of the slices with protein lysates from *control*, *junD*- and *Nfe2l2*-defcient fibroblasts.

Table S2: List of genes involved in oxidative stress response and up-regulated in low-*versus* high-H2AX TN breast tumours

Genes presented belong to the following gene ontology pathways: “response to stress” (P = 3.10^{-7}; Fold enrichment 2.1) and “NADPH oxidase complex” (P = 1.10^{-4}; Fold enrichment 35.7). These genes are significantly up-regulated in TN BC with low H2AX protein levels, compared to TN tumours with high H2AX protein levels. Subgroups of patients were defined by the median value of H2AX histological scores (Hscores) in the epithelial compartment. Differentially expressed genes were defined by Welch’s t test as indicated in the Methods section. BP = Biological Processes, CC = Cellular Components from the DAVID Gene Ontology analyses.

Table S3: **List of genes involved in oxidative stress response and up-regulated in low-*versus* high-H2AX HER2 breast tumours**

Genes presented belong to the following gene ontology pathways: “response to stress” (P = 3.10^{-11}; Fold enrichment 2.4), “superoxide anion generation” (P = 0.01; Fold enrichment 15.9), “response to oxidative stress” (P = 0.03; Fold enrichment 2.9) and “NADPH oxidase complex” (P = 0.006; Fold enrichment 24.2). Genes are significantly up-regulated in HER2 BC with low H2AX protein levels, compared to HER2 tumors with high H2AX protein levels. Subgroups of patients (Low- *versus* High-H2AX protein levels) were defined by the median value of H2AX histological scores (Hscores) in the epithelial compartment. Differentially expressed genes were defined by Welch’s t test as indicated in the Methods section. BP = Biological Processes, CC = Cellular Components from the DAVID Gene Ontology analyses.

Table S4: **List of genes involved in oxidative stress response and up-regulated after chemotherapy in TN breast tumours**

Genes presented belong to the following gene ontology pathways: “oxidation reduction” (P = 0.001; Fold enrichment = 2.1), “response to oxidative stress” (P = 0.02; Fold enrichment = 3.0) and “oxidoreductase activity” ($P = 5.10^{-4}$; Fold enrichment = 2.2). Genes are significantly up-regulated in TN BC after chemotherapy, compared to the corresponding biopsies before treatment. Differentially expressed genes per patient were defined by Paired t-test as indicated in the Methods section. BP = Biological Processes, MF = Molecular Functions from the DAVID Gene Ontology analyses.

Gruosso, Figure S1

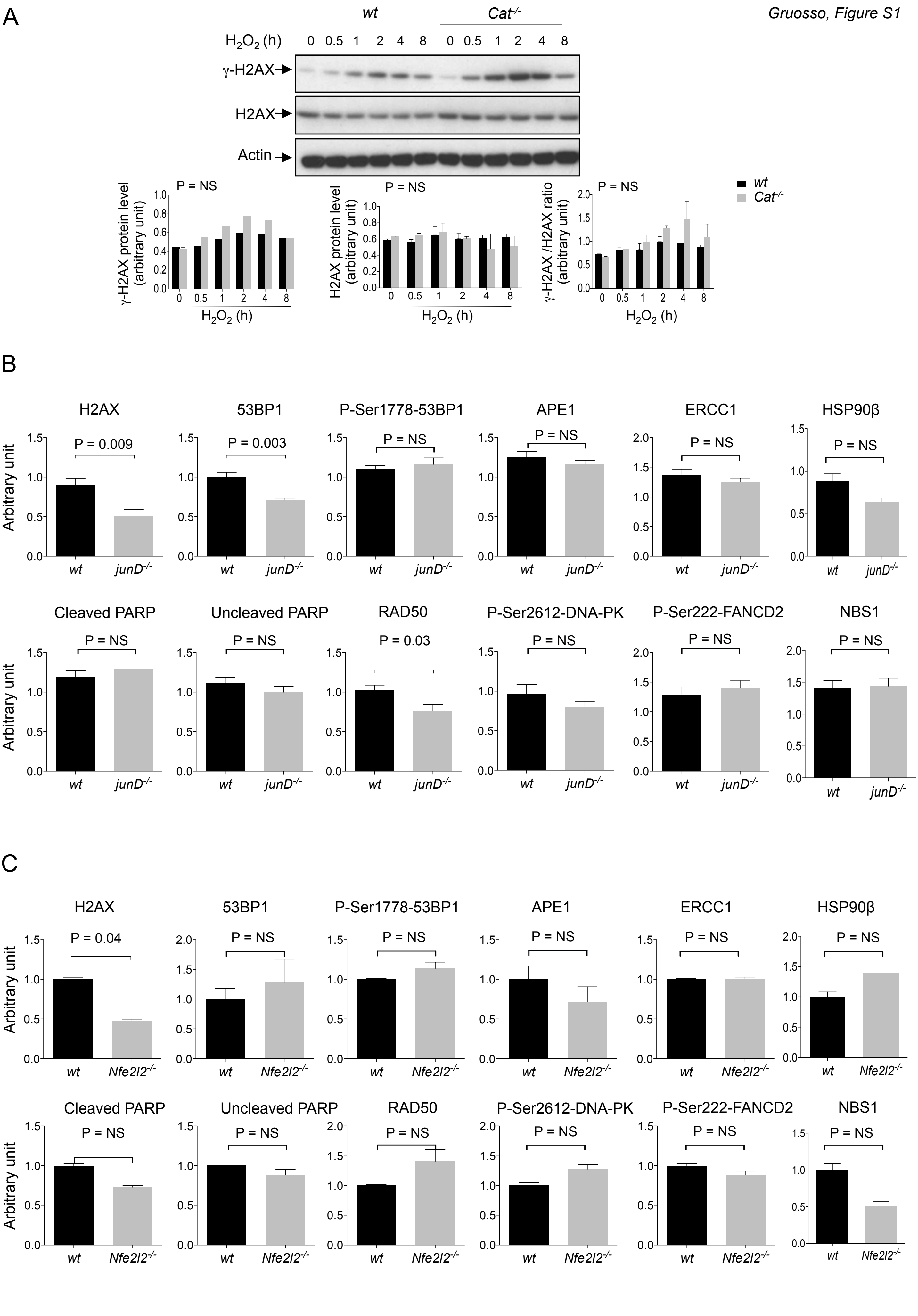

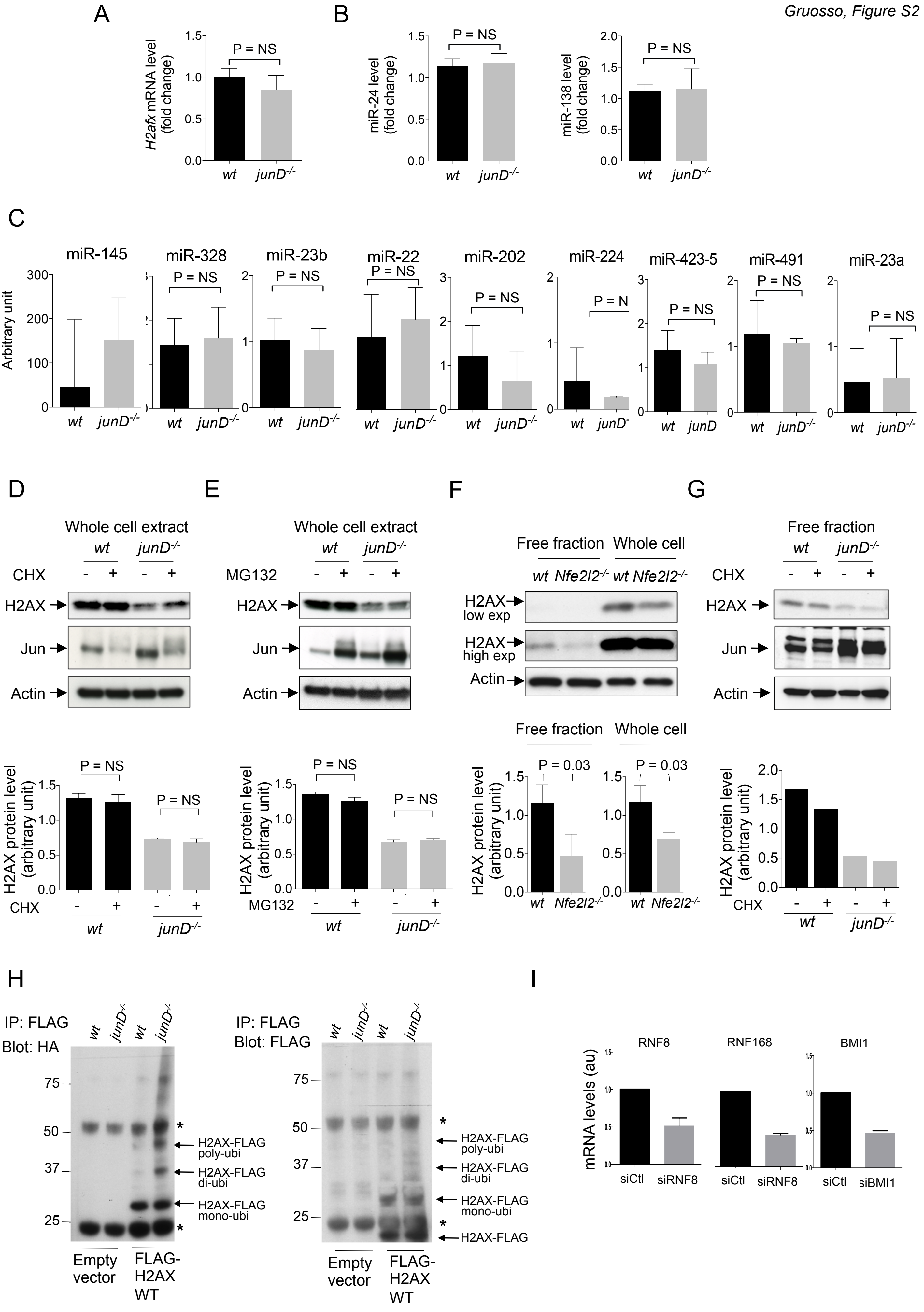
A
B
C
D
E
F
G
H
I
H2afx mRNA level (fold change)
miR-24 level (fold change)
miR-138 level (fold change)
P = NS
wt
junD-/-
Arbitrary unit
miR-145
miR-328
miR-23b
miR-22
miR-202
miR-224
miR-423-5
miR-491
miR-23a
Whole cell extract
CHX
MG132
H2AX
Jun
Actin
Free fraction
Whole cell
Nfe2l2-/-
low exp
high exp
H2AX protein level (arbitrary unit)
P = 0.03
IP: FLAG
Blot: HA
Blot: FLAG
H2AX-FLAG poly-ubi
H2AX-FLAG di-ubi
H2AX-FLAG mono-ubi
H2AX-FLAG
Empty vector
FLAG-H2AX WT
RNF8
RNF168
BMI1
mRNA levels (au)
siCtl
siRNF8
siBMI1

Name	Normalized Intensity values			p-value (adujsted t-test)	
	**wt**	**junD-/-**	**Nrf2-/-**	**p-value** (wt *vs* junD-/-)	**p-value** (wt *vs* Nrf2-/-)
ABI1(Phospho)	14,570169	19,183612	29,165935	NS	NS
ABL1	38,825043	115,352147	107,479713	NS	NS
ABL1(Phospho)	10,972764	13,825802	10,492690	NS	NS
AKT	11,068158	12,708612	12,858452	NS	NS
AKT1	19,059413	19,617469	11,765275	NS	NS
AKT1(Phospho)	17,051276	17,925411	23,762079	NS	NS
ATRIP(Phospho)	14,384715	7,989955	15,381367	NS	NS
BRCA1	17,387204	33,171339	44,121863	NS	NS
BRCA1(Phospho)	19,066176	16,649107	19,309036	NS	NS
c-Abl	20,429413	21,362641	21,839632	NS	NS
c-Abl(Phospho)	18,387843	17,412363	17,163460	NS	NS
CDC2(Phospho)	31,748177	33,040959	33,747773	NS	NS
CDC25A	12,274945	6,701064	13,831808	NS	NS
CDC25B	10,534465	4,394396	7,307709	NS	NS
CDC25B(Phospho)	18,846597	12,534784	7,545197	NS	NS
CDK1/CDC2	18,180933	19,823805	18,199311	NS	NS
CDK1/CDC2(Phospho)	18,838668	17,206566	8,656322	NS	0,0022
CDK2	14,709832	10,518644	35,513785	NS	0,0000
CDK2(Phospho)	15,890758	13,744068	11,041827	NS	NS
CDK7	18,316714	16,054018	15,400822	NS	NS
CDK7(Phospho)	12,135474	8,541636	32,926760	NS	0,0139
Chk1	16,495485	18,739432	18,884817	NS	NS
Chk2(Phospho)	17,906086	15,628904	26,894005	NS	0,0000
CyclinB1	18,569440	15,719304	17,463048	NS	NS
CyclinB1(phospho)	20,880737	18,701531	23,418518	NS	NS
CyclinD2	13,676759	18,546615	15,937898	NS	NS
CyclinD3(Phospho)	23,733063	22,864078	9,219745	NS	NS
CyclinE1	32,182571	60,303847	83,284836	NS	NS
CyclinE1(Phospho)	20,534070	21,786599	20,095733	NS	NS
CyclinE2	13,818017	16,822858	18,227561	NS	0,0171
DNA-PK	14,884427	17,300817	25,172397	NS	NS
DNA-PK(Phospho)	14,790438	15,005581	5,501996	NS	0,0000
E2F1(Phospho)	22,327201	10,910594	20,946396	NS	NS
E2F2(inter)	21,328324	23,098647	30,331192	NS	NS
E2F6(inter)	16,451407	21,075496	21,363292	NS	NS
Empty	1,380778	2,168422	2,019129	NS	NS
FKHR	20,022499	17,229830	13,580895	NS	NS
FKHR(Phospho)	19,907507	13,500409	19,991203	NS	NS
FKHRL1(Phospho)	15,580238	18,241868	30,595889	NS	0,0113
FOXO1/3/4-PAN	20,691806	23,819765	17,344806	NS	NS
FOXO1/3/4-PAN(Phospho)	23,431068	21,281794	30,445125	NS	0,0472
FOXO1A	16,707324	17,991352	8,827546	NS	0,0026
FOXO1A(Phospho)	17,627380	17,024495	27,307527	NS	NS
GAPDH	0,997305	4,307140	0,757822	NS	NS
GSK3a-b(Phospho)	17,282737	17,265690	8,132958	NS	0,0000
GSK3beta(Phospho)	21,030282	16,397667	31,505745	NS	0,0787
HDAC2	21,773814	17,492594	25,423131	NS	NS
HDAC2(Phospho)	19,107348	24,964956	32,292765	NS	0,0008
HDAC3(Phospho)	21,902094	18,829331	28,869829	NS	NS
HDAC4	10,268690	14,879784	10,884818	NS	NS
HDAC4(Phospho)	9,837225	21,353551	18,911452	NS	NS
HDAC5	14,633107	18,709081	13,297819	NS	NS
HDAC5(Phospho)	23,242756	14,110982	31,467551	NS	NS
HDAC6(Phospho)	13,374574	23,882008	30,964935	NS	NS
HDAC7(C-term)	16,296600	13,794256	7,427458	NS	0,0007
HDAC8	9,848155	10,299943	20,149720	NS	0,0000
HDAC8(Phospho)	19,496569	16,904322	25,476293	NS	0,0332
MDM2(Phospho)	21,181994	20,848553	23,416113	NS	0,0342
Myc	16,511130	12,034678	19,840476	NS	NS
MYT1	21,103792	23,140245	18,455860	NS	NS
P15INK(C-term)	23,809788	26,031572	28,090419	NS	NS
p18INK(inter)	18,960542	15,886006	6,303051	NS	0,0001
p21Cip1	8,511231	7,225121	18,802651	NS	0,0000
p21Cip1(Phospho)	18,088563	13,833004	3,569915	NS	0,0493
p27Kip1	10,667017	10,938018	3,750292	NS	NS
p27Kip1(Phospho)	18,966328	20,684393	26,569003	NS	NS
p300(N-term)	19,540170	14,124463	23,795462	NS	NS
p300/CBP(C-term)	17,478288	13,772571	7,489702	NS	0,0038
p53	17,453094	17,362522	18,835998	0,0501	0,0185
p53(Phospho)	23,156054	19,848071	28,948549	NS	0,0000
P90RSK	23,884584	37,759153	42,127265	NS	NS
PLK1	18,210295	24,965341	46,721990	NS	0,0000
PP2A-a(Phospho)	18,839430	25,078351	28,780412	NS	0,0345
RAD51	23,090116	27,093798	17,785538	NS	NS
RAD51(Phospho)	17,551441	18,837150	24,581872	NS	NS
RAD52(Phospho)	21,711115	18,826712	20,675417	NS	NS
Rb	15,886352	19,423881	21,352775	NS	0,0195
Rb(Phospho)	18,240418	14,054439	14,822179	NS	0,0438
Smad3	18,217153	20,750989	18,013800	NS	NS
Smad3(Phospho)	17,124858	20,246877	13,108144	0,0531	NS
Smad4(inter)	9,515085	7,133488	11,015730	NS	NS
SMC1	13,806944	14,833410	2,678488	NS	0,0004
SMC1(Phospho)	19,049745	10,612856	34,956858	NS	NS
TGFbeta1(inter)	19,128375	19,673627	16,904685	NS	NS
TGFbeta2(inter)	15,537899	27,759952	42,101492	NS	0,0004
TGFbeta3(inter)	16,722659	14,089028	6,715784	NS	0,0054
TGFbetareceptorII(inter)	12,809210	13,862239	18,773180	NS	NS
TGFBR1	19,839688	23,390838	14,991608	NS	NS
TOP2A/DNAtopoisomeraseII	21,925454	24,282317	26,794573	NS	NS
TopoisomeraseIIbeta(inter)	18,757585	17,193971	16,641029	NS	NS
WEE1(Phospho)	19,849094	17,716802	11,620901	NS	0,0093
14-3-3theta/tau(Phospho)	19,619800	20,686550	10,898745	NS	0,0000
14-3-3zeta	16,729351	12,287427	9,642600	NS	NS
14-3-3zeta(Phospho)	24,138238	18,375407	16,156002	NS	NS
14-3-3zeta/delta(Phospho)	19,747461	18,740819	22,746581	NS	NS

Gene Symbol	Gene name	Function	P	fold	included in
CD44	CD44 molecule	Receptor for collagens and matrix metalloproteinases involved in cell adhesion and migration.	0.004	2.0	BP
ARHGEF6	Rac/Cdc42 guanine nucleotide exchange factor 6	RhoGTPases that activates Rho proteins in many cellular processes.	0.004	1.9	BP
CYBA	cytochrome b-245, alpha polypeptide	Cytochrome b component 1 of 2, a component of the NADPH oxidase in phagocytes.	0.005	1.7	BP, CC
DUSP10	dual specificity phosphatase 10	Phosphatase that inactivates p38 and SAPK/JNK, but not MAPK/ERK.	0.005	1.6	BP
GADD45A	growth arrest and DNA-damage-inducible, alpha	Activation of the p38/JNK pathway following stressful growth arrest and DNA damage.	0.005	1.7	BP
NCF4	neutrophil cytosolic factor 4	Cytosolic regulatory component of the superoxide-producing phagocyte NADPH-oxidase.	0.006	2.2	CC
CXCR4	chemokine (C-X-C motif) receptor 4	CXC chemokine receptor specific for CXCL12/SDF1.	0.007	3.0	BP
FEN1	flap structure-specific endonuclease 1	Endonuclease processing overhanging flaps in DNA repair and Okazaki fragments.	0.007	1.6	BP
PDLIM1	PDZ and LIM domain 1	Adapter to bring other LIM-interacting proteins to the cytoskeleton.	0.007	1.5	BP
STAT3	signal transducer and activator of transcription 3	Transcription factor which plays a key role in cell growth and apoptosis after stress stimuli.	0.009	1.6	BP
B4GALT1	beta 1,4-galactosyltransferase,peptide 1	Enzyme that participates both in glycoconjugate and lactose biosynthesis.	0.01	1.6	BP
CD38	CD38 molecule	Multifunctional enzyme inmplicated in cell adhesion and signalling.	0.01	3.7	BP
NFX1	nuclear transcription factor, X-box binding 1	Transcriptional repressor that may regulate the duration of an inflammatory response.	0.01	1.5	BP
SLAMF7	SLAM family member 7 (also known CS1)	Isoform1 mediates NK cell activation through ERK-mediated pathway.	0.01	2.3	BP
BCL3	B-cell CLL/lymphoma 3	Transcriptional co-activator involved in the regulation of NF-kappa B activation.	0.02	1.7	BP
CCL18	chemokine (C-C motif) ligand 18	Chemotactic activity for naive T cells, CD4+ and CD8+ T cells and nonactivated lymphocytes.	0.02	3.8	BP
CCL2	chemokine (C-C motif) ligand 2	CXC cytokine that has chemotactic activity for monocytes and basophils.	0.02	2.0	BP
C5AR1	complement component 5a receptor 1	Signal transducer in inflammation, particularly in IL12 and TNFA production in monocytes.	0.02	1.6	BP
CYBB	cytochrome b-245, beta polypeptide	Cytochrome b component 2 of 2, a component of the NADPH oxidase inphagocytes.	0.02	1.9	BP, CC
LYZ	lysozyme	Anti-microbial agent whose natural substrate is the bacterial cell wall peptidoglycan.	0.02	4.6	BP
MAP3K13	mitogen-activated protein 3(kinase) 13	Activation of MAPK8/JNK, MAP2K7/MKK7, which suggests a role in the JNK signaling pathway.	0.02	1.7	BP
NCF1	neutrophil cytosolic factor 1	Cytosolic subunit of neutrophil NADPH oxidase that produces superoxide anion.	0.02	1.9	BP
NCF1C	neutrophil cytosolic factor 1C pseudogene	NCF1 pseudogene unlikely to express a protein.	0.02	1.8	BP, CC
POLH	polymerase (DNA directed), eta	DNA polymerase which replicates UV-damaged DNA ; suppressing its mutagenic effect.	0.02	1.5	BP
RAD51L1	RAD51 homolog B	Binds to RAD51C and is involved in the homologous recombination DNA repair.	0.02	2.1	BP
SERPINA3	serpin peptidase inhibitor clade A member 3	Plasma protease inhibitor that can inhibit convertases of angiotensin-1 into active angiotensin-2	0.02	5.9	BP
SOD2	superoxide dismutase 2, mitochondrial	Antioxidant, superoxide radicals dismutation into hydrogen peroxide and oxygen	0.02	1.8	BP
SOCS3	suppressor of cytokine signaling 3	Cytokine-inducible negative regulator of cytokine signaling which inhibits JAK2.	0.02	1.9	BP
VNN1	vanin 1	Pantetheinase which may play a role in oxidative-stress response.	0.02	1.9	BP
BCL6	B-cell CLL/lymphoma 6	Transcription repressor involved in B lymphocyte maturation	0.03	1.6	BP
BRIP1	BRCA1 interacting protein C-terminal helicase 1	RecQ DEAH helicase important for the double-strand break repair function of BRCA1.	0.03	2.0	BP
CD40	CD40	TNF receptor involved in immune and inflammatory response.	0.03	1.7	BP
ERN1	endoplasmic reticulum to nucleus signaling 1	Endoribonuclease which modulates gene expressionin response to ER stress.	0.03	1.5	BP
ERRFI1	ERBB receptor feedback inhibitor 1	Shares homology with a protein product induced during cell stress including growth	0.03	1.9	BP
FADS1	fatty acid desaturase 1	Fatty acid desaturase regulating unsaturation of fatty acids.	0.03	1.6	BP
RAB27A	RAB27A, member RAS oncogene family	Membrane protein involved in protein transport and small GTPase mediated signal transduction.	0.03	1.6	BP
SGK3	serum/glucocorticoid regulated kinase 3	Role in neutral amino acid transport and activation of potassium and chloride channels.	0.03	1.7	BP
TRD@	T cell receptor delta locus	T cell receptor delta chain.	0.03	2.0	BP
CDH3	cadherin 3, type 1, P-cadherin (placental)	Calcium-dependent cell-cell adhesion glycoprotein.	0.04	1.9	BP
C1S	complement component 1, s subcomponent	Serine protease in the human complement subcomponent C1.	0.04	2.6	BP
C3	complement component 3	Activation of complement system againt bacterial infection.	0.04	1.8	BP
IL27RA	interleukin 27 receptor	Regulation of Th1-type immune responses via STAT3 and STAT1.	0.04	2.5	BP
JAG2	jagged 2	One of several ligands that activate Notch and related receptors.	0.04	1.6	BP
TLR1	toll-like receptor 1	Cooperates with TLR2 to mediates immune response to bacterial via NF-kappa-B activation.	0.04	2.3	BP
DERL3	derlin 3	Degradation of misfolded glycoproteins in the ER.	0.05	2.0	BP
FANCA	Fanconi anemia, complementation group A	DNA repair protein involved in interstrand DNA cross-link repair and chromosome stability	0.05	1.7	BP
SAA1	serum amyloid A1	Amyloid A precursor involved in HDL metabolism and cholesterol metabolism.	0.05	2.0	BP
SAA2	serum amyloid A2	Amyloid A precursor involved in HDL metabolism and cholesterol metabolism.	0.05	2.0	BP
TLR8	toll-like receptor 8	Participated in immune response to bacterial lipoproteins via NF-kappa-B activation.	0.05	2.2	BP

Gene Symbol	Gene name	Function	P	fold	included in
IL1RN	interleukin 1 receptor antagonist	IL1A and IL1B inhibitor which modulates immune and inflammatory responses	0.002	3,2	BP
MANF	mesencephalic astrocyte-derived neurotrophic factor	Involved in resistance to ER stress-induced death and cell proliferation	0.002	1,6	BP
C5AR1	complement component 5a receptor 1	Stimulates chemotaxis, granule enzyme release and superoxide anion production	0.004	1,9	BP
NOD2	nucleotide-binding oligomerization domain 2	Protein involved in immune responseto intracellular bacteria	0.004	2,0	BP
VSIG4	V-set and immunoglobulin domain containing 4	Receptor for complement fragments and potentially a negative regulator of T-cell responses	0.004	3,5	BP
AQP9	aquaporin 9	Membrane channels which stimulates urea transport and osmotic water permeability	0.005	1,8	BP
SIGLEC1	sialic acid binding Ig-like lectin 1,sialoadhesin	Lectin-like adhesion molecule that mediates cell-cell interactions	0.006	2,7	BP
RBM3	RNA binding motif	RNA-binding protein induced by cold shock and low oxygen tension	0.007	1,8	BP
SYT7	synaptotagmin VII	Synaptotagmin that mediates calcium-dependent regulation of membrane trafficking	0.007	1,6	BP
NCF2	neutrophil cytosolic factor 2	NADPH oxidase subunit that generates reactive oxidant intermediates	0.008	2,7	BP, CC
CTSB	cathepsin B	Lysosomal cysteine proteinase	0.01	1,6	BP
CYBA	cytochrome b-245,alpha polypeptide	Cytochrome b component 1 of 2, a component of the NADPH oxidase in phagocytes	0.01	2,0	BP, CC
MMP14	matrix metallopeptidase 14 (membrane-inserted)	Involved in the breakdown of extracellular matrix in normal physiological processes	0.01	1,5	BP
LYN	v-yes-1 Yamaguchi sarcoma oncogene homolog	Maybe involved in the regulation of mast cell degranulation, and erythroid differentiation	0.011	2,0	BP
AKR1B1	aldo-keto reductase family 1,member B1	Catalyzes the reduction of a carbonyl-containing compounds to their corresponding alcohols	0.02	1,7	BP
CCR1	chemokine (C-C motif) receptor 1	Chemokine receptor involved in inflammation	0.02	1,7	BP
CD163	CD163 molecule	Receptor that protects tissues from free hemoglobin-mediated oxidative damage	0.02	2,5	BP
DERL3	derlin 3	Involved in the degradation of misfolded glycoproteins in the ER	0.02	1,6	BP
HRH1	histamine receptor H1	Messenger molecule released from mast cells, enterochromaffin-like cells, and neurons	0.02	1,9	BP
HSPA5	heat shock 70kDa protein 5	Folding, assembly and transport of proteins in the ER	0.02	1,3	BP
KCNJ8	potassium channel,subfamily J,member 8	Potassium channels	0.02	1,8	BP
LBP	lipopolysaccharide binding protein	Acute-phase immunologic response to gram-negative bacterial infections	0.02	6,0	BP
NCF4	neutrophil cytosolic factor 4	NADPH oxidase subunit that generates reactive oxidant intermediates	0.02	1,8	CC
PAPSS2	3'-phosphoadenosine 5'-phosphosulfate synthase 2	Bifunctional enzyme involved in the sulfate activation pathway	0.02	2,3	BP
PKN1	protein kinase N1	Activated by Rho small G proteins and mediates the Rho-dependent signaling pathway	0.02	1,6	BP
PROCR	protein C receptor,endothelial	Receptor for activated protein C involved in the blood coagulation pathway	0.02	1,5	BP
SERPINE1	serpin peptidase inhibitor,clade E,member 1	Inhibitor of fibrinolysis	0.02	2,1	BP
TGFB1	transforming growth factor,beta 1	Multifunctionnal cytokine involved in proliferation, differentiation, adhesion and migration	0.02	1,7	BP
ARHGEF6	Rac/Cdc42 guanine nucleotide exchange factor 6	RhoGTPases that activates Rho proteins in many cellular processes	0.03	1,8	BP
C3AR1	complement component 3a receptor 1	Stimulates chemotaxis, granule enzyme release and superoxide anion production	0.03	1,7	BP
CD14	CD14 molecule	Participates in the innate immune response to bacterial lipopolysaccharide	0.03	2,2	BP
CD40	CD40 molecule	TNF receptor involved in immune and inflammatory response	0.03	1,5	BP
CD44	CD44 molecule	Receptor for collagens and metalloproteinases involved in cell adhesion and migration	0.03	1,5	BP
CLU	clusterin	Secreted chaperone that can under some stress conditions also be found in the cell cytosol	0.03	2,1	BP
FCGR1A	Fc fragment of IgG receptor	Related immunoglobulin gamma Fc receptor gene A	0.03	2,2	BP
IGFBP2	insulin-like growth factor binding protein 2	Inhibits IGF-mediated growth and developmental rates	0.03	2,9	BP
LAMB2	laminin,beta 2	Membranes constituent involved in cell adhesion, migration, signaling and metastasis	0.03	2,1	BP
PDPN	podoplanin	May be involved in cell migration and/or actin cytoskeleton organization	0.03	1,6	BP
PLA2G4C	phospholipase A2,group IVC	Involved in production of signaling molecules	0.03	1,5	BP
PLA2G7	phospholipase A2,group VII	Degradation of platelet-activating factor to biologically inactive products	0.03	2,6	BP
PLEK	pleckstrin	Major protein kinase C substrate of platelets	0.03	1,9	BP
SLC11A1	solute carrier family 11 member 1	Metal transporter involved in iron metabolism and host resistance to certain pathogens	0.03	2,0	BP
SLC1A3	solute carrier family 1,member 3	Transports glutamate andaspartate and act as a symport by cotransporting sodium	0.03	1,8	BP
TLR1	toll-like receptor 1	Cooperates with TLR2 to mediate immune response to bacterial via NF-kappa-B activation	0.03	1,9	BP
TLR2	toll-like receptor 2	Cooperates with TLR1 to mediate immune response to bacterial via NF-kappa-B activation	0.03	2,1	BP
ANXA1	annexin A1	Phospholipase inhibitory activity involved in exocytosis and probably anti-inflammatory	0.04	2,1	BP
C1QA	complement component 1,q subcomponent,A chain	C1q subcomponent, itself component of the serum complement system	0.04	2,3	BP
C1QB	complement component 1,q subcomponent,B chain	C1q subcomponent, itself component of the serum complement system	0.04	2,3	BP
FANCI	Fanconi anemia,complementation group I	Involved in homologous recombination and in interstrand DNA cross-links repair	0.04	1,7	BP
FCGR1C	Fc fragment of IgG receptor,pseudogene	Related immunoglobulin gamma Fc receptor gene C	0.04	2,3	BP
GPX1	glutathione peroxidase 1	Antioxydant enzyme that detoxifies hydrogen peroxide	0.04	1,7	BP
MX1	myxovirus resistance 1 p78	Interferon-inducible protein responsible for an antiviral state against influenza virus	0.04	2,3	BP
MX2	myxovirus resistance 2	Member of both the dynamin family and the family of large GTPases	0.04	1,9	BP
PTAFR	platelet-activating factor receptor	Platelet-activating factor receptor that stimulates signal transduction pathways	0.04	1,6	BP
PTGS1	prostaglandin-endoperoxide synthase 1	Conversion of arachinodate to prostaglandin involved in angiogenesis	0.04	1,8	BP
SOD2	superoxide dismutase 2	Antioxidant, superoxide radicals dismutation into hydrogen peroxide and oxygen	0.04	1,7	BP
TNFSF4	tumor necrosis factor superfamily,member 4	Ligand for TNFRSF4/OX4 and is involved in T cell antigen-presenting cell interactions	0.04	1,8	BP
TRPA1	transient receptor potential cation channel,A,1	May be involved in signal transduction and growth control	0.04	2,3	BP
CXCL16	chemokine ligand 16	Binds to oxidized low density lipoprotein and induces chemotactic and calcium mobilization	0.05	2,0	BP
ENTPD1	ectonucleoside triphosphate diphosphohydrolase 1	Hydrolyzes ATP and ADP	0.05	1,6	BP
ERO1L	ERO1-like (S. cerevisiae)	Oxidoreductase that oxidizes in the endoplasmic reticulum, generating oxidative stress	0.05	1,7	BP

Gene Symbol	Gene name	Function	P	fold	included in
DUSP1	dual specificity phosphatase 1	Phosphatase involved in cellular response to stress and cell proliferation	0.002	4.1	BP
FAR2	fatty acyl CoA reductase 2	Reductase enzyme involved in fatty acids into fatty alcohols	0.003	1.3	BP, MF
HSD17B7P2	hydroxysteroid (17-beta) dehydrogenase 7 pseudogene 2	Dehydrogenase implicated in de novo cholesterol synthesis	0.004	1.2	MF
DHCR7	7-dehydrocholesterol reductase	Conversion of 7-dehydrocholesterol to cholesterol	0.01	1.3	BP, MF
FOS	FBJ murine osteosarcoma viral oncogene homolog	Transcription factor in signal transduction, proliferation and transcriptomic response to stress	0.01	4.0	BP
GPD2	glycerol-3-phosphate dehydrogenase 2	Reoxidization of NADH formed during glycolysis	0.01	1.2	BP, MF
GFER	growth factor, augmenter of liver regeneration	Homolog of yeast scERV1 essential for oxidative phosphorylation	0.02	1.2	BP, MF
PCYOX1	prenylcysteine oxidase 1	Degradation of prenylcysteine to yield free cysteines and a hydrophobic isoprenoid product	0.02	1.2	BP, MF
RRM2B	ribonucleotide reductase M2 B	Reduction of ribonucleoside diphosphates into deoxyribonucleoside diphosphates	0.02	1.2	BP, MF
ATRN	attractin	Secreted chemotactic protein in inflammatory responses and energy homeostatis	0.03	1.2	BP
CYP27A1	cytochrome P450, family 27, subfamily A, polypeptide 1	Monooxygenases in the bile synthesis pathway	0.03	1.4	BP, MF
GCLM	glutamate-cysteine ligase, modifier subunit	First rate limiting enzyme of glutathione synthesis	0.03	1.4	BP, MF
HPGD	hydroxyprostaglandin dehydrogenase	Alcohol dehydrogenase involved in the metabolism of prostaglandins	0.03	1.7	BP, MF
ME1	malic enzyme 1	NADPH generation for fatty acid biosynthesis by oxidative decarboxylation of malate	0.03	1.6	BP, MF
OLR1	oxidized low density lipoprotein receptor 1	Scavenger receptor involved in oxidized low density lipoprotein degradation	0.03	2.1	BP
SOD1	superoxide dismutase 1	Antioxidant, superoxide radicals dismutation into hydrogen peroxide and oxygen	0.03	1.1	BP, MF
AIFM2	apoptosis-inducing factor, mitochondrion-associated, 2	Oxidoreductase that binds single stranded DNA	0.04	1.2	BP, MF
CRYL1	crystallin, lambda 1	L-gulonate dehydrogenation in the uronate cycle, an alternative glucose metabolic pathway	0.04	1.2	MF
EPAS1	endothelial PAS domain protein 1	Transcription factor involved in the induction of oxygen regulated genes	0.04	1.4	BP
GCDH	glutaryl-CoA dehydrogenase	Oxidative decarboxylation in the degradative of lysine and tryptophan metabolism	0.04	1.2	BP, MF
HSD17B7	hydroxysteroid (17-beta) dehydrogenase 7	Biosynthesis of sex steroids and a reductase in the biosynthesis of cholesterol	0.04	1.2	BP, MF
LOX	lysyl oxidase	Crosslinking of collagens and elastin by catalyzing oxidative deamination	0.04	1.5	BP, MF
RETSAT	retinol saturase	Retinol saturase carrying out the saturation of all-trans-retinol	0.04	1.3	BP, MF
SLC25A13	solute carrier family 25 member 13	Exchange of aspartate for glutamate and a proton across the inner mitochondrial membrane	0.04	1.3	BP
SOD3	superoxide dismutase 3	Antioxidant, superoxide radicals dismutation into hydrogen peroxide and oxygen	0.04	1.2	BP, MF
TP53I3	tumor protein p53 inducible protein 3	Similar to oxidoreductases implicated in response to oxidative stress and irradiation	0.04	1.2	BP, MF
AKR1A1	aldo-keto reductase family 1, member A1	Aldehyde reductase involved in the reduction of biogenic and xenobiotic aldehydes	0.05	1.1	BP, MF
ALDH1A1	aldehyde dehydrogenase 1 family, member A1	Cytosolic aldehyde dehydrogenase in the major pathway of alcohol metabolism	0.05	1.3	BP, MF
POR	P450 (cytochrome) oxidoreductase	Endoplasmic reticulum membrane oxidoreductase	0.05	1.2	BP, MF
