# Supplementary figures and images for: Chronic oxidative stress promotes H2AX protein degradation and enhances chemosensitivity in breast cancer patients
Source: EMBO Mol Med. 2016 Mar 22;8(5):527–49. doi: 10.15252/emmm.201505891 (PMC5123617; doi:10.15252/emmm.201505891)

D

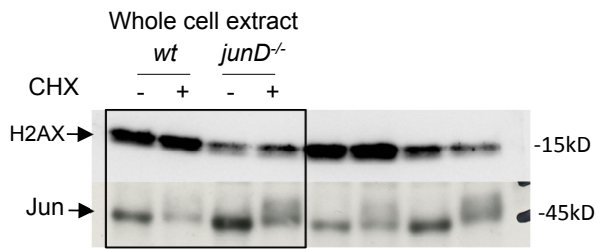

E

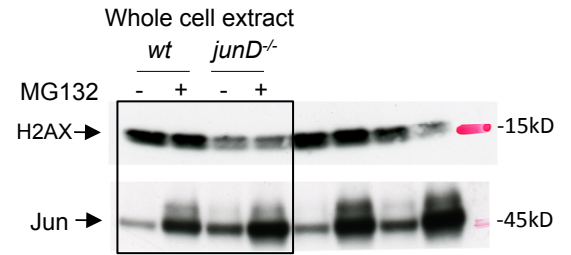

F

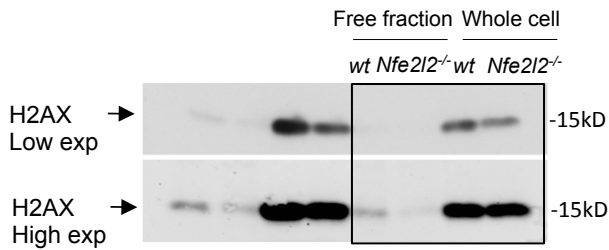

G

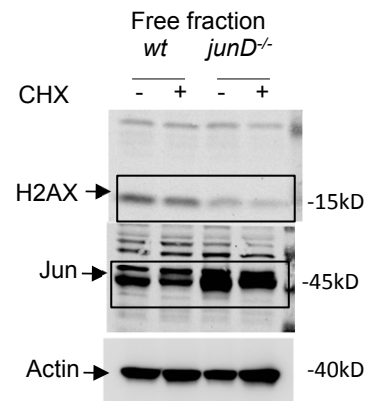

H

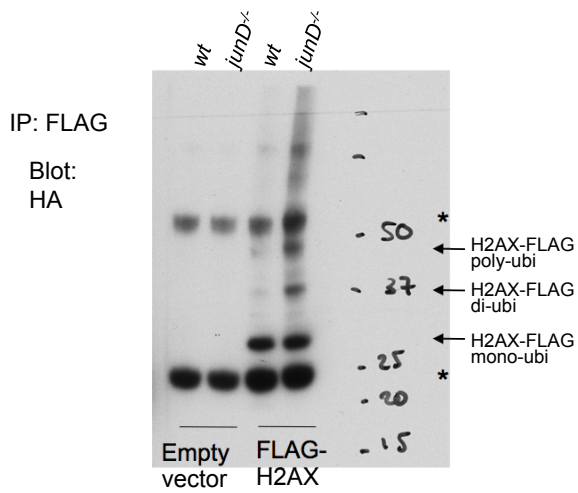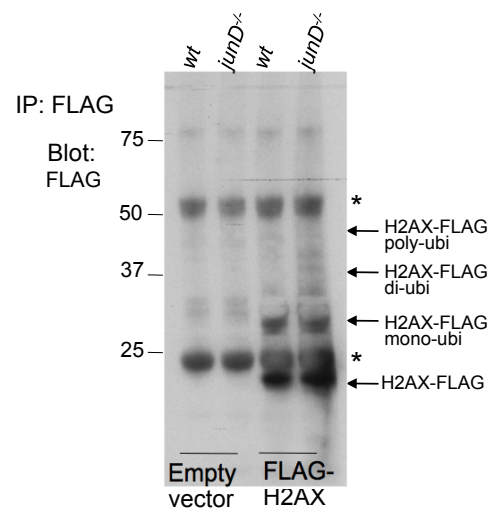

Supplement: Supplementary file 2 — Source Data for Appendix [file EMMM-8-527-s004.zip › Source_data_for_Appendix_Figures/Source_data_for_Appendix_Figure_2.pdf]

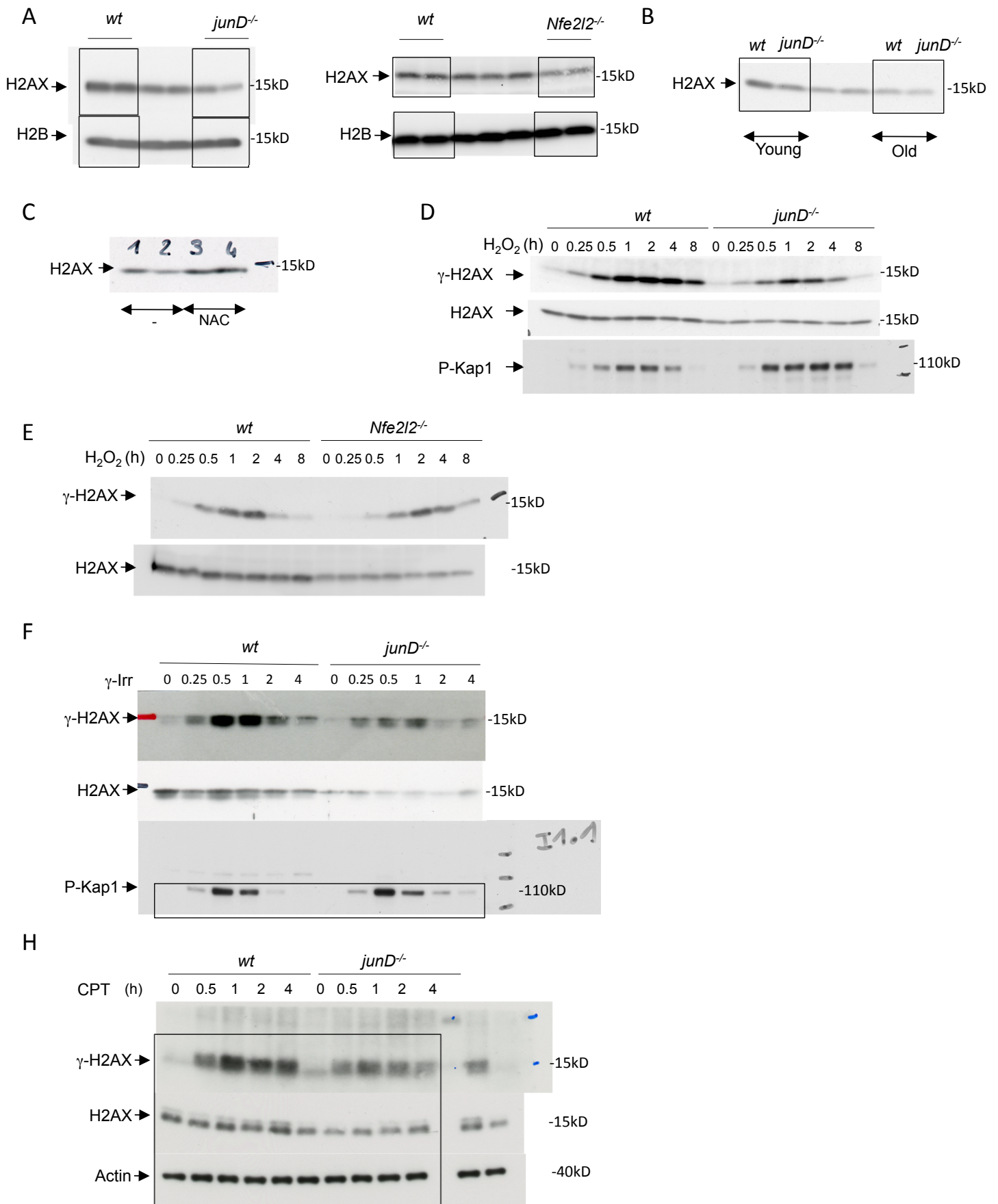

Supplement: Supplementary file 4 — Source Data for Figure 1 [file EMMM-8-527-s002.pdf]

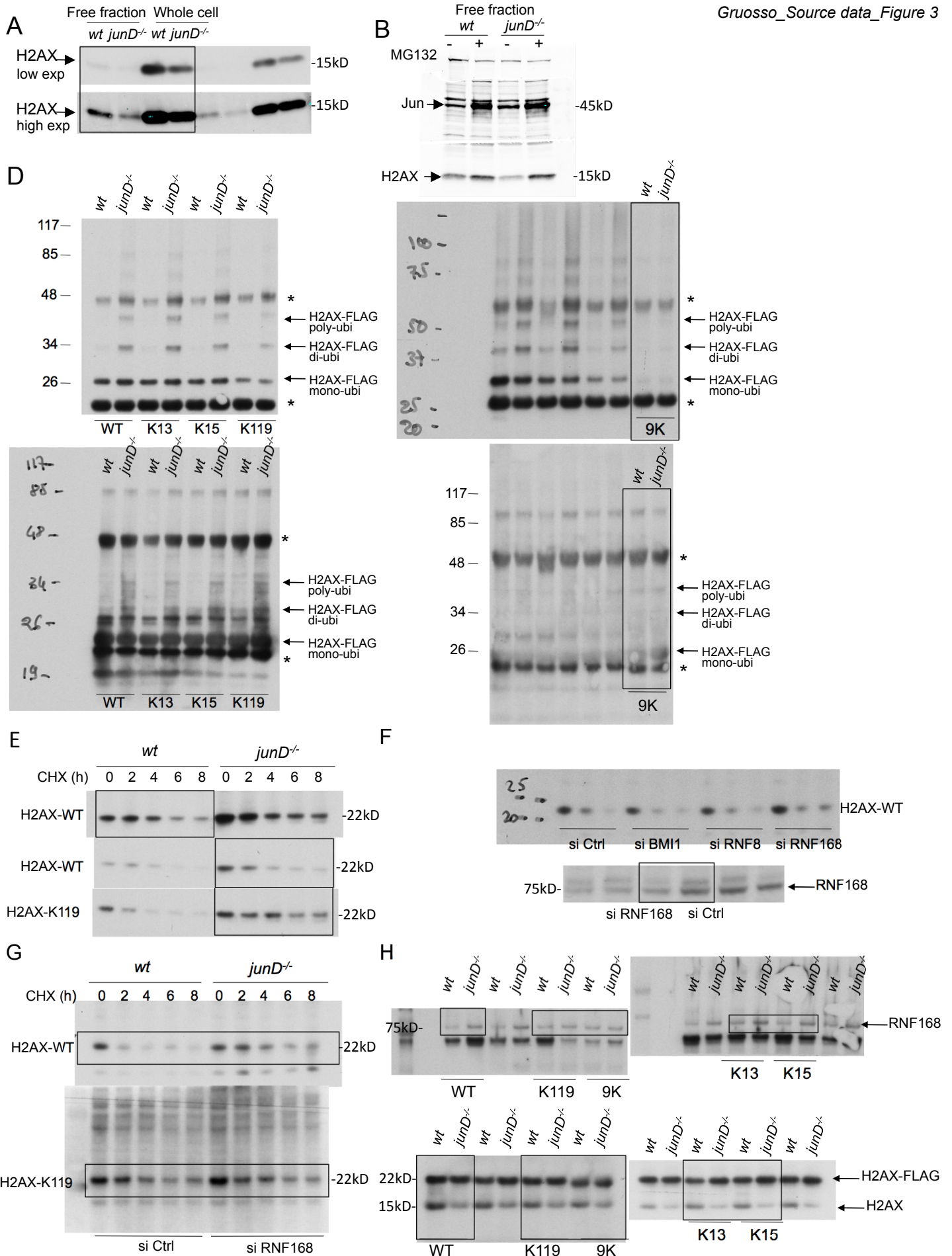

Supplement: Supplementary file 5 — Source Data for Figure 3 [file EMMM-8-527-s003.pdf]
