# Supplementary material for: Chronic oxidative stress promotes H2AX protein degradation and enhances chemosensitivity in breast cancer patients
Source: EMBO Mol Med. 2016 Mar 22;8(5):527–49. doi: 10.15252/emmm.201505891 (PMC5123617; doi:10.15252/emmm.201505891)
Supplement: Supplementary file 2 — Source Data for Appendix [file Source_data_for_Appendix_Figure_1.pdf › Source_data_for_Appendix_Figures/Source_data_for_Appendix_Figure_1.pdf]

A

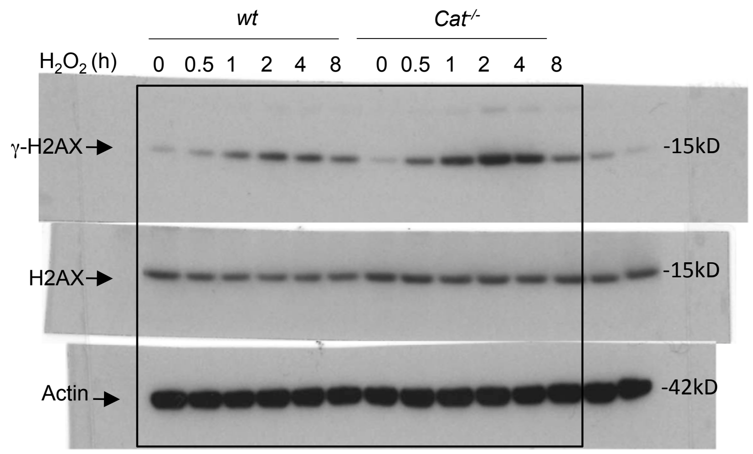
wt
Cat-/-
H2O2 (h)
0 0.5 1 2 4 8 0 0.5 1 2 4 8
γ-H2AX
-15kD
H2AX
-15kD
Actin
-42kD
